# Evolution of cooperativity in the spin transition of an iron(II) complex on a graphite surface

**DOI:** 10.1038/s41467-018-05399-8

**Published:** 2018-07-30

**Authors:** Lalminthang Kipgen, Matthias Bernien, Sascha Ossinger, Fabian Nickel, Andrew J. Britton, Lucas M. Arruda, Holger Naggert, Chen Luo, Christian Lotze, Hanjo Ryll, Florin Radu, Enrico Schierle, Eugen Weschke, Felix Tuczek, Wolfgang Kuch

**Affiliations:** 1 Institut für Experimentalphysik, Freie Universität Berlin, Arnimallee 14, 14195 Berlin, Germany; 2 Institut für Anorganische Chemie, Christian-Albrechts-Universität zu Kiel, Max-Eyth-Straße 2, 24118 Kiel, Germany; 3 Institut für Experimentelle and Angewandte Physik, Universität Regensburg, Universitätsstrasse 31, 93053 Regensburg, Germany; 4 Helmholtz-Zentrum Berlin für Materialien und Energie, Albert-Einstein-Straße 15, 12489 Berlin, Germany

## Abstract

Cooperative effects determine the spin-state bistability of spin-crossover molecules (SCMs). Herein, the ultimate scale limit at which cooperative spin switching becomes effective is investigated in a complex [Fe(H_2_B(pz)_2_)_2_(bipy)] deposited on a highly oriented pyrolytic graphite surface, using x-ray absorption spectroscopy. This system exhibits a complete thermal- and light-induced spin transition at thicknesses ranging from submonolayers to multilayers. On increasing the coverage from 0.35(4) to 10(1) monolayers, the width of the temperature-induced spin transition curve narrows significantly, evidencing the buildup of cooperative effects. While the molecules at the submonolayers exhibit an apparent anticooperative behavior, the multilayers starting from a double-layer exhibit a distinctly cooperative spin switching, with a free-molecule-like behavior indicated at around a monolayer. These observations will serve as useful guidelines in designing SCM-based devices.

## Abstract (web summary)

Spin-crossover molecules offer a potential route towards molecular spintronics, but retaining the bistability of the spin state upon surface deposition is challenging. Here, the authors study the spin-crossover behaviours of an Fe(II) complex deposited on graphite, determining the scale limit at which cooperative spin switching becomes effective.

## Introduction

Spin-crossover molecules (SCMs) offer a tantalizing route towards the realization of molecular spintronics^1–3^ and other nanoscale devices^4–7^ due to their bistability in the spin state, which can be manipulated or switched reversibly with external stimuli like temperature, light, pressure, electric field, magnetic field, or x rays^8–12^. The spin-crossover phenomenon in SCMs stems from the competition between the mean spin-pairing energy (Δ), which favors the high-spin (HS, paramagnetic) state and the ligand field—quantified by the ligand-field parameter 10*Dq*—which favors the low-spin (LS, diamagnetic or less paramagnetic than HS) state. Recent experiments with single molecules, thin films, and nanoparticles of SCMs revealed a rich and interesting interplay between the transport properties and the molecular spin states: high conductance in the HS state and low conductance in the LS state^11,13–15^, accompanied by the generation of spin-polarized current depending upon the spin state of the molecule and the electrodes^16^, and memory effects in the room-temperature regime^17–19^.

To put things into perspective, besides SCMs, the family of bistable molecules with a comparable promise of technological spin-offs are single-molecule magnets (SMMs)^20–22^. SCMs, however, offer one distinct advantage over SMMs: whereas SMMs exhibit bistability only at low temperatures^23^, room-temperature regimes can be reached for SCMs^24–27^. Nevertheless, reports of SCMs on surfaces are relatively scarce. This can be traced back to two main reasons: the paucity of SCMs that are vacuum-evaporable, with only a few cases reported so far^27–38^, and the coexistence of the two spin states or the loss of spin-crossover behavior altogether on contact with surfaces. It appears to be rather the norm that SCMs in contact with a surface invariably lead to the co-existence of the HS and the LS states at all temperatures^36,39–44^, though a few exceptions exist^33,45^.

In a study of [Fe(phen)_2_(NCS)_2_] on a Cu surface, spin-state coexistence in a series of submonolayer coverages has been reported, while molecules decoupled from the substrate (bilayer) exhibit a dominant spin state^39^. In the same system, electrically induced spin switching has been reported with the introduction of a thin insulating spacer layer of CuN between the molecules and the substrate^13^. In our previous studies, we have shown that for the SCMs [Fe(H_2_B(pz)_2_)_2_(phen)]^40^ and [Fe(H_2_B(pz)_2_)_2_(phen-me_4_)]^36^ (an analog of the former) in contact with a Au surface, the molecules undergo fragmentation, while only in the second layer, the molecules’ integrity, as well as the spin-crossover are preserved. However, on a Bi surface, even though the molecules’ integrity is preserved already in the first layer, about half of the molecules are locked in the HS state^36^. In a bilayer of [Fe(H_2_B(pz)_2_)_2_(bipy)] (hereafter referred to as Fe(bpz)-bipy) on Au probed with temperature-dependent scanning tunneling microscopy (STM), spin-state coexistence has been reported while the composition of the spin states was independent from temperature^46^. This led to the speculation of the spin-state coexistence to be an intrinsic property of SCMs in ultrathin films^46^. In a recent STM-based study of a monolayer of [Fe^II^((3,5-(CH_3_)_2_Pz)_3_BH)_2_] on a Au surface, the authors reported a collective behavior in the light-induced HS→LS transition at 4.6 K, albeit with both spin-states coexisting^43^. Owing to the observed long-range order of alternating HS and LS states^43^, it is likely that the molecules undergo an antagonistic or anticooperative spin transition.

The absence or presence of cooperativity in the spin transition of SCMs and their characteristics is one of the most important issues for research, since it is responsible for the bistability in the spin states of SCMs. SCMs in the bulk generally exhibit cooperativity while undergoing the spin transition, which is ascribed to elastic interactions arising from the volume difference between the HS state (larger volume) and the LS state (smaller volume)^47,48^. The strength of the cooperative effects can be inferred from the degree of steepness of the temperature-induced spin transition curve. The interactions that favor “like-spin” pairings (HS–HS or LS–LS) are termed cooperative (ferromagnetic-like), while those that favor “unlike-spin” pairings (HS–LS) are termed anticooperative (antiferromagnetic-like). Determining the ultimate scale limit at which cooperativity becomes effective is of major interest when aiming at applications at reduced size or dimensionality. Future devices will likely involve integrating multi-functional molecules like SCMs with 2D materials exhibiting novel properties. In this regard, graphene is among the material of choice due to its robust optical, electrical and mechanical properties^49^. Understanding the behavior of SCMs on a highly oriented pyrolytic graphite (HOPG) surface will be an ideal platform towards integrating it with graphene.

Herein, we report on the complete and efficient thermal- and light-induced spin-crossover of Fe(bpz)-bipy on an HOPG surface with coverages ranging from 0.35(4) to 10(1) ML (monolayers) investigated by x-ray absorption spectroscopy, while addressing two main traits: the nature of the spin-crossover behavior for molecules in direct contact with the surface as well as upon increasing the coverage of the molecular layers, and the metastability of the HS state on the surface after it is switched from the LS state by illumination with a green LED (light emitting diode, *λ* = 520 nm) at low temperatures. Our results reveal the evolution of cooperativity in the spin-crossover of ultrathin molecular layers of SCMs adsorbed on a solid surface, which is evident from an increased steepness in the thermal spin-crossover curves. (The degree of steepness or the transition-width (Δ*T*) is defined as the temperature difference at which 80% of the molecules are in the HS and LS states, respectively^45^.) The interaction strength, deduced from fits to the phenomenological Slichter and Drickamer mean-field model^50^, increases monotonically with the coverage, reaching about 60% of the bulk value at a coverage of 10(1) ML. On the other hand, the HS→LS relaxation measurements of submonolayer coverages at 8, 20, 30, and 40 K show a stretched exponential behavior of the amount of HS molecules as a function of time, which may be explained by assuming anticooperativity at submonolayer coverages.

## Results

### Temperature- and light-induced spin-crossover

**Fig. 1:**
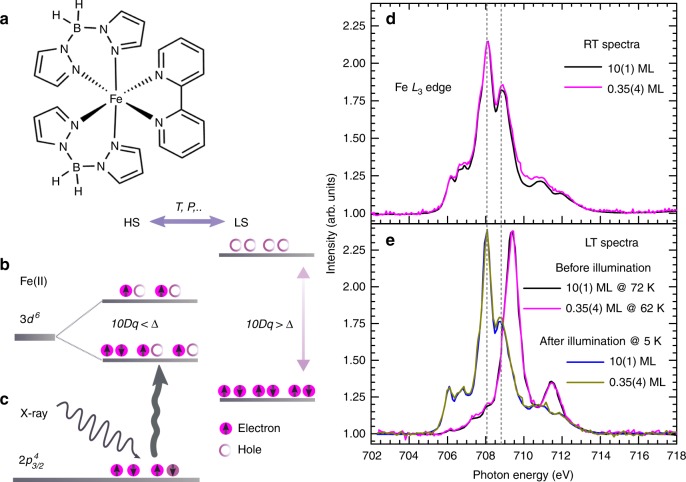
High-spin and low-spin states of spin-crossover molecules as revealed by x-ray absorption spectroscopy. **a** Chemical structure of Fe(bpz)-bipy; **b** The size of the ligand field splitting of the Fe 3*d*^6^ orbital into e_*g*_ and t_2*g*_ (10*Dq*) relative to the mean spin-pairing energy (Δ) leads to the HS or LS state; **c** Interaction of x rays with $2{\mathrm{p}}_{{\mathrm{3/2}}}^{\mathrm{4}}$ electrons and their excitation to the 3*d* orbital (Fe *L*_3_ edge); **d** RT Fe *L*_3_ absorption spectra of 10(1) ML and 0.35(4) ML of Fe(bpz)-bipy on HOPG (black and magenta lines, respectively), and **e** at 72 K and 62 K before illumination (black and magenta lines, respectively), and after illumination with a green LED (blue and dark-yellow lines, respectively) at 5 K. The absorption intensities of the 0.35(4)-ML sample have been scaled to that of the 10(1)-ML sample

To probe the spin state of the molecules, x-ray absorption spectroscopy (XAS) is used. XAS is very well suited for studying SCMs on surfaces due to its element selectivity and high sensitivity in tracing a subtle electronic or chemical change^51^. More specifically, the Fe *L*_2,3_ (2p^6^3d^6^→2p^5^3d^7^) or the Fe *L*_3_ $\left( {2{\mathrm{p}}_{{\mathrm{3/2}}}^{\mathrm{4}}3{\mathrm{d}}^6 \to 2{\mathrm{p}}_{{\mathrm{3/2}}}^{\mathrm{3}}3{\mathrm{d}}^7} \right)$ edge spectral line shape is a fingerprint of the magnetic state of the molecule^52^. Schematic representations of the 3*d*-orbital electronic distributions of Fe(bpz)-bipy (Fig. 1a) in the HS and the LS states, and transitions from the $2{\mathrm{p}}_{{\mathrm{3/2}}}^{\mathrm{4}}$ core level causing the Fe *L*_3_ absorption edge are shown in Fig. 1b, c, respectively. It should be noted as a word of caution that the x-ray beam can induce LS→HS or HS→LS transitions at low temperatures, a process termed as soft x-ray-induced excited spin state trapping (SOXIESST)^12^, and reverse-SOXIESST^53^, respectively. The x-ray-induced effects on the spin states can be largely mitigated by maintaining the photon flux in the range of ~10^9^ photons s^−1^ mm^−2^^ 53^ (cf. Methods).

Figure 1d, e shows the Fe *L*_3_ spectra of 0.35(4) and 10(1) ML at RT (room temperature) and at LT (low temperature) before and after illumination with a green LED. The RT and LT spectra before illumination have sharply contrasting Fe *L*_3_ line shapes: the RT spectrum is characterized by two main peaks at 708.1 and 708.9 eV and the LT spectrum by a single main peak at 709.4 eV before illumination with light. These spectral line shapes have been established as characteristic of the HS and the LS states, respectively^33^^,^^45^^,^^52^. The spectrum at any other intermediate temperature is a linear combination of the two (cf. Supplementary Figure 1), as has also been shown elsewhere^33^^,^^45^. Upon illumination with the green LED at 5 K, the RT spectral line shape is recovered albeit with higher XA intensity (LS→HS transition). The molecules are thus again in the HS state, which is termed light-induced excited spin-state trapping (LIESST)^9^. The bulk Fe *L*_3_ spectra recorded at RT and at LT also yielded correspondingly similar line shapes to the ones described above (cf. Supplementary Figure 1). While SOXIESST is saturated at about 60% HS with an x-ray photon flux of about ~1 × 10^9^ photons s^−1^ mm^−2^ ^53^, the final states in LIESST are assumed to be 100% HS following ref.^45^, where the spectral profiles can be reproduced by spin-multiplet calculations. On warming up to 70–90 K, the samples can be converted back to the LS state. To test the reversibility in the spin switching during the cooling and heating cycles, systematic measurements to track the change in Fe *L*_3_ spectra (and hence the change in the spin state) have been carried out between 5 and 96 K under continuous illumination, for the 0.69(8)-ML sample as proof of concept. This is shown in Supplementary Figure 2: the sample exhibits a near-complete reversible spin transition from HS to LS state during ramp-up and LS to HS state during ramp-down in the aforementioned temperature range.

**Fig. 2:**
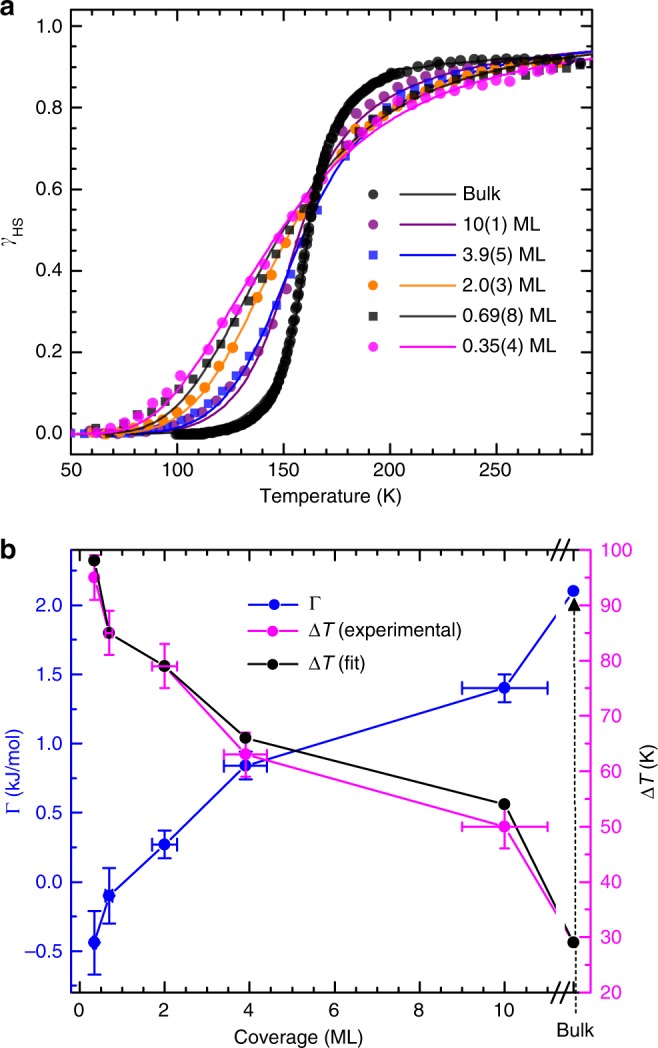
Temperature-dependent spin-crossover curves as signatures of cooperative effects. **a** Temperature-dependent spin-crossover of different coverages of Fe(bpz)-bipy on HOPG along with that of the bulk data; the dots represent experimental data while the solid lines are fits obtained from the Slichter–Drickamer model. **b** Dependence of the interaction parameter Γ (left-axis) and the transition width Δ*T* (right-axis) upon the coverage indicating the inverse relation between the two. The bulk data are taken from Moliner et al.^54^

The fraction of HS molecules (*γ*_HS_) at any given temperature is estimated by fitting the corresponding spectrum to that of a linear combination of the RT Fe *L*_3_ XA spectrum (HS state) and the LT spectrum (LS state) recorded at low temperature before the onset of LIESST or SOXIESST. The low-temperature HS and LS spectra across all the coverages have similar line shapes upon scaling, though the HS spectra at RT show some minor variations which appeared as about 3% change in the spin-state compositions (c.f. Supplementary Figure 1). For uniformity, the RT spectrum of 10(1) ML is taken as the reference HS state for all the coverages, with its *γ*_HS_ assumed to be 0.91 in conformity with that reported for the bulk molecule^54^; this number is also reproduced by using the LT spectrum after LIESST (100% HS) as the reference HS state. The presence of a certain quantity of LS molecules at RT in thin films of Fe(bpz)-bipy – similar to the bulk – has also been reported elsewhere^31^. The temperature-dependent measurements of *γ*_HS_ are carried out by cooling the sample at the rate of 4 K min^−1^ while simultaneously recording the spectra. The result is shown in Fig. 2a, and is characterized by a gradual to a relatively steeper spin transition when going from submonolayer to multilayer coverages, indicating an increase in cooperativity in the molecular spin switching processes.

Traditionally, elastic interactions between the molecules have been invoked to rationalize the occurrence of cooperativity: The spin transition is concomitantly accompanied by a change in volume (expansion and contraction in the HS and LS states, respectively), which induces elastic strain, resulting in both short- and long-range interactions between the molecules^47,55^. However, a recent wave-function ab initio calculation suggests the cooperativity to arise mainly from electrostatic contributions as a result of the simultaneous electronic relocalization within the molecules and fluctuations in the Madelung field during the spin transition^56,57^. Herein, we choose to treat the interaction between the molecules in contact with the HOPG surface and at higher coverages by the classical thermodynamic model of Slichter and Drickamer (S−D model)^50^. In this model, a term Γ(1 − *γ*_HS_)*γ*_HS_ is introduced in the expression of Gibb’s free energy, where Γ is a phenomenological interaction parameter. The macroscopic S−D model is similar to the microscopic two-level Ising-like model in the mean-field approach^58^. A model based on interacting HS and LS domains (as opposed to HS and LS states) still yields a Gibb’s free energy similar to the S–D model^59^. Wavefunction ab initio calculations also reproduced the S–D model^57^, which has been used recently to explore the possibility of an enhanced cooperativity in surface-supported 2D metal-organic frameworks^60^. At equilibrium, the S−D model leads to the implicit equation:

1 $${\mathrm{ln}}\left( {\frac{{1 - \gamma _{\mathrm{HS}}}}{{\gamma _{\mathrm{HS}}}}} \right) = \frac{{{\mathrm{\Delta }}H + \Gamma (1 - 2\gamma _{\mathrm{HS}})}}{{RT}} - \frac{{{\mathrm{\Delta }}S}}{R}{\kern 1pt} $$

where Δ*H* and Δ*S* are the differences in enthalpy and entropy, respectively, between the HS and LS states; *R* is the universal gas constant. The experimental input in Equation (1) is the HS fraction *γ*_HS_. Of the three fitting parameters, namely, Γ, Δ*H* and Δ*S*; Δ*H* and Δ*S* are related by the transition temperature *T*_1/2_ (defined as the temperature where the population of the HS and the LS species are equal), as Δ*H* = *T*_1/2_⋅Δ*S*. If Γ = 0, then Equation (1) reduces to the van’t Hoff’s model – a non-interacting molecular system.

The results obtained by a fit of the experimental data by the S–D model using the method of least-square deviation are presented in Table 1 together with bulk data taken from Moliner et al.^54^. It yields a gradual evolution in cooperative spin transition in going from submonolayers to multilayers: negative interaction parameters at 0.35(4) (Γ = −0.44(0.23) kJ mol^−1^) and 0.69(8) ML (Γ = −0.1(2) kJ mol^−1^), positive at 2.0(3) ML (Γ = 0.3(1) kJ mol^−1^), and further increase with increasing coverage. In contrast, the entropy change Δ*S* and the enthalpy change Δ*H* across all coverages yield roughly constant values. The result for Γ is plotted in Fig. 2b as a function of coverage (blue data points, left axis). The interaction parameter Γ and the transition width Δ*T*, also shown in Fig. 2b, show an inverse relation. The similar values of Δ*T* obtained from the experimental data and the S−D model fit across all coverages (Fig. 2b right axis, magenta and black dots) are an indication of the suitability of the model in tracing the evolution of cooperativity in the spin-transition processes in ultrathin films. The details of the S–D model fits are given in Supplementary Figures 3–5 and Supplementary Table 1.

**Table 1:** Thermodynamic parameters

Coverage (ML)	Δ*T*(K)		Γ (kJ mol^−1^)	Δ*S* (J mol^−1^ K^−1^)	Δ*H* (kJ mol^−1^)	*T*_1/2_ (K)	
0.35(4)	95(4)^a^	98^b^	−0.44(0.23)	43(3)	6.4(4)	150(2)^a^	148.5^b^
0.69(8)	85(4)	85	−0.1(2)	44(3)	6.6(5)	153(2)	150
2.0(3)	79(4)	79	0.3(1)	44(1)	6.7(2)	154(2)	154
3.9(5)	63(4)	66	0.8(1)	44(2)	7.0(3)	162(2)	160
10(1)	50(4)	54	1.4(1)	39(2)	6.2(3)	160(2)	158
Bulk^*c*^	29	27	2.1	47.4	7.7	159.5	162

The 80-to-20% width of the temperature-dependent spin-crossover curve, the interaction parameter, the entropy and enthalpy differences between the HS and LS states, and the transition temperature

^a^ Experimental data; ^b^ Determined from best fit of the model; ^c^ Bulk data taken from ref.^54^. The uncertainties of Γ, Δ*H*, and Δ*S* given in parentheses are the standard errors as obtained from the fit of the non-linear model. The uncertainties of Δ*T* and *T*_1/2_ are estimated from the scattering of the data points

**Fig. 3:**
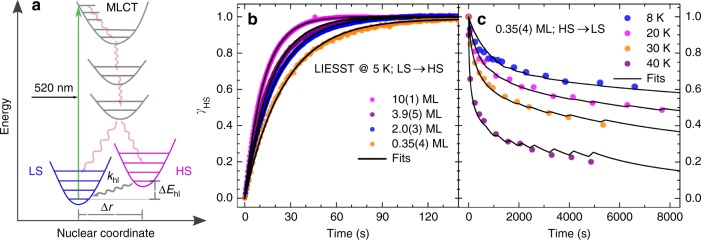
Light-induced LS→HS state transition and the eventual HS→LS state decay at low temperatures. **a** Schematic representation of the light-induced LS→HS state transition—the excitation of electrons from the LS state to the MLCT (metal-to-ligand charge transfer) states, and subsequent relaxation to the HS state via intermediate states—and the eventual decay of HS to the LS state; **b** light-induced LS → HS state transition at 5 K for the different coverages, and **c** the HS→LS relaxation of 0.35(4) ML at 8, 20, 30, and 40 K (dots). The solid lines are the result of a simultaneous fits to Equation (2) together with the SOXIESST rates. The step-like features at the data points are due to the SOXIESST (soft-x-ray-induced excited spin-state trapping) terms

The time-dependent population of the HS state upon illumination with a green LED for all the coverages at 5 K is shown in Fig. 3b. The process, known as LIESST, has been rationalized in terms of intersystem crossing^48^ (the scheme is shown in Fig. 3a) and has been probed in detail with ultrafast optical and x-ray spectroscopies^61^. Because the light-induced LS→HS transition is quite fast, the kinetics of this effect cannot be measured by taking complete absorption spectra. Instead, it was determined by recording the time dependence of the absorption signal with the x-ray energy fixed at 708.1 eV (the intensity of the Fe *L*_3_ spectrum at this energy above the background is proportional to the HS content), while keeping the illumination on. The complete Fe *L*_3_ XA spectra recorded before and after such a timescan are used to ascertain the conversion of the spin states (cf. Supplementary Figure 6).

The HS fraction as a function of illumination time is obtained by normalization of the recorded timescan signal between 0 (the LS state) and 1 (the HS state). Fitting the LS→HS transition with a single exponential function yields rate constants of 0.065(1), 0.0501(1), 0.0583(2), and 0.0823(1) s^−1^ for 0.35(4), 2.0(3), 3.9(5), and 10(1) ML, respectively. However, these rate constants consist of LIESST, as well as SOXIESST components; the latter arising from the x-ray exposure during the timescan. In order to separate the two, one can use the x-ray-induced LS→HS (SOXIESST) and HS→LS (reverse-SOXIESST) transition rate constants reported in the same system (0.8 ML of Fe-(bpz)bipy on HOPG) at the same temperature (5 K), which are 3.50(3) × 10^−4^ s^−1^ and 2.3(1) × 10^−4^ s^−1^, respectively^53^. The rate constants solely from LIESST are then estimated as 0.0369(1), 0.0505(1), 0.0589(2), and 0.0827(1) s^−1^ for 0.35(4), 2.0(3), 3.9(5), and 10(1) ML, respectively. With the green LED photon flux density of 4.2(8) × 10^14^ photons s^−1^ mm^−2^ (c.f. Methods), the corresponding effective cross sections are estimated as 0.009(2), 0.012(2), 0.014(3), and 0.019(4) Å^2^, respectively. Since each of the curves can be fitted by a mono-exponential function albeit with different rate constants, it is concluded that the light-induced LS→HS transition arises from an individual molecule–photon interactions without any role of cooperative effects. This result is in agreement with LIESST in bulk SCMs, where it is also found to be a single–molecule phenomenon^48^. The observed efficient switching of the spin states with light in these SCMs in contact with an HOPG surface can be exploited for applications in optical memory and display elements^4^.

### Stability of light-induced HS state at low temperatures

Figure 3c shows the results of HS→LS relaxation measurements of 0.35(4) ML at 8, 20, 30, and 40 K. The measurements are done by firstly switching the sample to the HS state by green-LED illumination. With the illumination off, the subsequent spin relaxation is traced from Fe *L*_3_ spectra recorded as a function of time. The low-temperature HS→LS relaxation process is characterized by an initial fast relaxation and then slows down with time, leading to a stretched exponential. This is in sharp contrast to the bulk, where the HS state is rather stable in the temperature range considered here, and only in the thermally activated regime (>40 K), the spin relaxation becomes pronounced and exhibits sigmoidal characteristics^54^. The HS → LS relaxation observed on the surface can be modeled with a phenomenological equation involving a negative interaction parameter *α*:

2 $$\frac{{\partial \gamma _{\mathrm{HS}}}}{{\partial t}} = - k_{hl}{\kern 1pt} \gamma _{\mathrm{HS}}\,{\mathrm{exp}}\left[ {\alpha \left( {1 - \gamma _{\mathrm{HS}}} \right)} \right]$$

where *k*_*hl*_ denotes the HS → LS relaxation rate constant. Equation (2) is similar to the phenomenological model introduced by Hauser to explain the HS → LS relaxation in the bulk SCMs, but with *α* replaced by *α*/*T* in the thermally activated regime so as to account for the sigmoidal-type HS → LS relaxation behavior^62^. For an accurate modeling of the HS→LS relaxation, the x-ray-induced spin transitions occuring during the data acquisition, namely, *k*_1_ · (1 − *γ*_HS_) for LS→HS and *k*_2_·*γ*_HS_ for HS→LS transitions have to be included in Equation (2)^53^. A simultaneous fit of Equation (2) to the spin relaxation measurements at all temperatures including the SOXIESST terms yields the value of the interaction parameter *α* to be −6.3(3). The relaxation measurements of 0.69(8) ML show a behavior similar to that of 0.35(4) ML (cf. Supplementary Figure 7). A comparison of ln(*k*_*hl*_(*T*)) for both coverages is also given in Supplementary Figure 7. In comparison to the 0.69(8)-ML sample, the metastable HS state of the 0.35(4)-ML sample decays much more rapidly to the ground state (LS state) in the temperature range between 8 and 30 K (cf. Supplementary Figure 7). However, at 40 K, the relaxation rates become similar: 0.025(5) and 0.021(7) s^−1^ for the 0.35(4) and 0.69(8)-ML sample, respectively. In the bulk relaxation data provided in ref.^54^, the relaxation rate at 42 K is about three orders of magnitude lower than that of the submonolayer samples at 40 K. These differences in the decay rates might arise either from enhanced tunneling rates or a reduction in the energy barriers between the HS and LS wells; or a combination of both, in the submonolayer samples as compared to the bulk (the quantum tunneling rate is related to the characteristic vibrational frequency of the [FeN_6_] core^62^).

## Discussion

For SCMs in the bulk, the interaction parameters *α* and Γ are related by a constant, *α* = *p*Γ^62^. The constant *p* is inversely proportional to the characteristic vibrational frequency of the [FeN_6_] core and has a weaker dependence upon the vertical displacement as well as the horizontal displacement of the LS and HS wells through the reduced energy and the Huang-Rhys factor, respectively, which enter logarithmically^62^. The horizontal displacement is proportional to the Fe–N bond-length difference between the HS and LS states while the reduced energy is the ratio of the vertical displacement (Δ*E*_*hl*_, Fig. 3(a), the zero-point energy difference between the HS and LS wells) to the characteristic vibrational quanta of the [FeN_6_] core. Using the values of the parameters given in ref.^54^, *p* is estimated to be 1.3 × 10^−3^ J^−1^ mol for Fe(bpz)-bipy in the bulk. For the 0.35(4)-ML sample and assuming that the same relation still holds (given the negative values of both *α* and Γ), a value of *p* = 1.4(9) × 10^−2^ J^−1^ mol is obtained. The higher value of *p* by about an order of magnitude in the 0.35(4)-ML sample as compared to the bulk might be attributed to a reduced characteristic vibrational frequency of the [FeN_6_] core in the former, possibly due to molecule–substrate interactions. Alternatively, a distribution in the energy barriers between HS and LS states – arising from disorder or conformational flexibility of the ligand – can also result in a stretched exponential HS→LS relaxation, as reported in the case of some bulk SCMs exhibiting strong cooperativity in the temperature-induced spin transition, but nevertheless displaying a stretched-exponential spin relaxation due to such factors^63,64^. It is therefore not clear whether the stretched exponential decay results exclusively from an antagonistic (or anticooperative) spin transition. Regardless of the mechanism, it should be mentioned that this is the first report on the HS→LS relaxation of SCMs on a surface exhibiting a stretched exponential decay.

The buildup of cooperativity in molecular layers of only a few monolayers thickness indicates the presence of intermolecular interactions across the molecular layers. While a two-dimensional arrangement such as submonolayer islands exhibits an apparent antagonistic behavior arising either from interactions that favor unlike-spin states or from a distribution in the energy barriers between the two spin states, clear signs of cooperative spin switching are observed starting already from the second layer. The further increase in the degree of cooperativity with increasing thickness could be related to the higher coordination of molecules in the inner layers compared to the surface layers, the reduction in the relative amount of surface or interface molecules, and/or the reduced importance of molecules in direct contact with the substrate. It is interesting to compare the results presented here with spin-crossover nanoparticles, although the direct external environments of SCMs on the surface and those of nanoparticles are different in that the nanoparticles are always coated with a stabilizer which acts as a rigid matrix. Nevertheless, spin-crossover nanoparticles are also found to exhibit a gradual temperature-dependent spin-transition like the one observed here in the case of ultrathin films, but with the transition temperatures being proportional to the particles’ sizes. However, at particle sizes of less than 10 nm, hysteretic behavior (memory effects) appears – which have been attributed to an increase in the lattice stiffness leading to greater cooperative effects^65^. The presence (or absence) of hysteretic behavior in these ultrathin films of Fe(bpz)-bipy is not known, as the spin-state change in the opposite direction, i.e., temperature ramp-up from low- to room-temperature, has not been measured. It is worth noting that a small hysteresis of about 4 K has been reported in a relatively thick vacuum-deposited film (355 nm) of Fe(bpz)-bipy^31^, despite the absence of such a behavior in the bulk^54,66^. To the best of our knowledge, however, for vacuum-deposited films with thickness in the range of our samples – maximum thickness of about 12 nm – the presence of hysteresis has never been reported.

In summary, we have shown the complete thermal- and (highly efficient) light-induced spin-crossover of Fe(bpz)-bipy on the HOPG surface of coverages ranging from 0.35(4) ML to 10(1) ML. While a free-molecule-like spin-crossover around a monolayer is indicated, cooperativity is clearly evident in the double layer and at higher coverages as revealed by the transition width of the thermal-induced spin-crossover curves. The light-induced LS→HS state switching, on the other hand, is independent of cooperative effects. These findings are relevant for nanoscale applications relying on spin-state bistability.

## Methods

### Sample preparation

The SCM Fe(bpz)-bipy is synthesized according to the procedure reported by Real et al.^66^. The molecular powder is evaporated from a tantalum Knudsen cell at 160 °C at a pressure of ~2 × 10^−9^ mbar, and deposited onto the HOPG substrate held at room-temperature. The evaporation rate is controlled from the frequency change of a quartz crystal attached to the Knudsen cell. The HOPG substrate (ZYA) of dimension 12 × 12 × 2 mm^3^ and mosaic spread angle of 0.4(1)° is purchased from Structure Probe. The cleaving of the HOPG substrate with a carbon tape is performed (so as to obtain a clean surface) in a loadlock chamber maintained at ~10^−7^ mbar connected to the sample preparation chamber. The molecular coverage is estimated from integrated peak intensity of the Fe *L*_3_ spectrum and frequency shifts of the quartz crystal which is integrated with the evaporator. The details of the coverage estimation procedure are provided in Supplementary Figure 8 and Supplementary Notes 1.

### X-ray absorption spectroscopy

The XAS measurements are performed in-situ at the high-field diffractometer of the beamline UE46-PGM1 and at the VEKMAG end-station of the beamline PM2 of BESSY II at a pressure of about 5 × 10^−11^ mbar and 1.5 × 10^−10^ mbar, respectively. The photon flux at the UE46-PGM1 beamline is reduced by a factor of about 15 by damping the beam with an Al-foil of thickness ~3 μm, such that the estimated photon flux is ~1 × 10^9 ^photons s^−1^ mm^−2^; this is done in order to mitigate the soft-x-ray-induced excited spin state trapping (SOXIESST) at low temperatures^53^. At the PM2 beamline, the photon flux is estimated to be ~1.6 × 10^9^ photons s^−1^ mm^−2^. The data shown in Figs. 1e, 2, 3b originates from the high-field diffractometer end-station, while the data shown in Fig. 3c was measured at the VEKMAG end-station. All spectra are recorded by means of total electron yield, where the sample drain current is recorded as a function of the x-ray photon energy and normalized to the photocurrent of a Au grid (a Pt grid in PM2-VEKMAG^67^) upstream to the experiment, and to the background signal from a clean HOPG substrate. The Fe *L*_3_ spectra are recorded at the magic angle 54.7° between the surface and the *k* vector of the linearly *p*-polarized x rays. At this angle, the XA resonance intensities are independent of the orientations of the molecular orbitals. The measurements involving light-induced effects at low temperatures were performed with a green LED of *λ* = 520 nm with a spectral width (fwhm) of 30 nm. The flux density at the sample position is estimated as 4.2(8) × 10^14^ photons s^−1^ mm^−2^. The details of the optical setup have been described elsewhere^45^.

### Atomic force microscopy

In order to ascertain the efficacy in the deposition of the Fe(bpz)-bipy on the HOPG surface, atomic force microscopy (AFM) images of a series of submonolayer coverages have been recorded ex situ at room temperature. One such image is shown in Supplementary Figure 9; under ambient conditions, the molecules form nanoporous islands, quite similar to the structure of a submonolayer of [Fe(H_2_B(pz)_2_)_2_(phen)] on the same surface^45^. The line profile reveals an island height in the range of 1.0 nm, which is consistent with the height of a molecule. This is true for all the submonolayer coverages recorded. Although the morphology of the molecular islands in vacuum may be different, one can conclude that no 3D crystallites are formed and that all the molecules are in contact with the surface. The AFM (Nanotec Cervantes) measurements are carried out ex situ in ambient conditions in tapping mode using a Si cantilever of stiffness 2.7 N/m with a resonance frequency of 75 kHz.

### Data Availability

All the relevant data can be obtained from the authors on reasonable request.

## Electronic supplementary material

Supplementary Information

Peer Review
