# Supplementary material for: Evolution of cooperativity in the spin transition of an iron(II) complex on a graphite surface
Source: Nat Commun. 2018 Jul 30;9:2984. doi: 10.1038/s41467-018-05399-8 (PMC6065309; doi:10.1038/s41467-018-05399-8)
Supplement: Supplementary file 1 — Supplementary Information [file 41467_2018_5399_MOESM1_ESM.pdf]

Supplementary Information for

Evolution of Cooperativity in the Spin Transition of an Iron(II) Complex on a Graphite Surface

Kipgen *et al.*

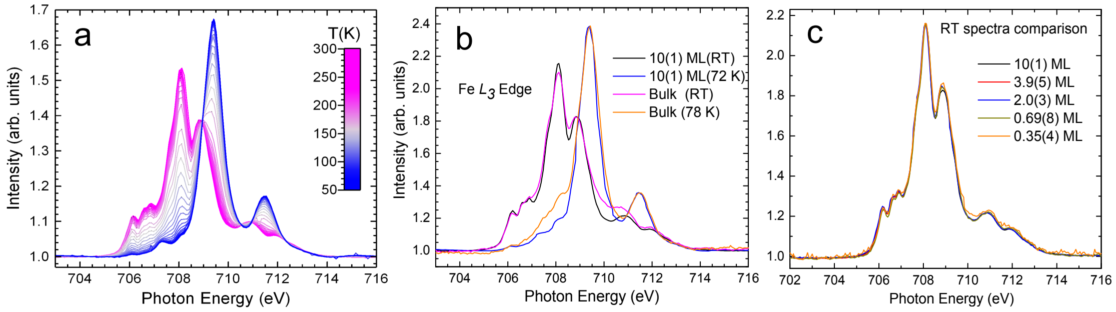

Supplementary Figure 1: Temperature-dependent Fe L_3 x-ray absorption spectra. (a) Temperature-dependent Fe L_3 XA spectra of the 3.9(5)-ML sample recorded while cooling from room temperature to 50 K at a rate of 4 K·min^{-1}. The Fe L_3 x-ray absorption (XA) spectral profile is a fingerprint of the spin state of spin-crossover molecules (SCMs): the room temperature (RT) spectrum is characterized by the high-spin state (HS, S = 2) and the low temperature (LT) spectrum in the region of 50–80 K by the low-spin state (LS, S = 0). The spectrum at any other intermediate temperature is a linear combination between the two; (b) Comparison of the room- and low-temperature Fe L_3 spectra of the 10(1)-ML sample and the bulk. The bulk sample is prepared by crimping the molecular powder onto an indium foil. There are some minor variations between the RT and LT spectra of the ultrathin film and the bulk — probably due to the different background contributions to the XA signal from the substrates (HOPG in the former and indium foil in the latter) — and not necessarily reflecting differences in the HS or the LS contents; and (c) RT Fe L_3 spectral comparison of different coverages prepared on HOPG. The line shapes are very similar, indicating the uniformity in the HS (or the LS) composition across all the samples at RT. The XA intensities in (b) and (c) are scaled to that of the 10(1)-ML sample.

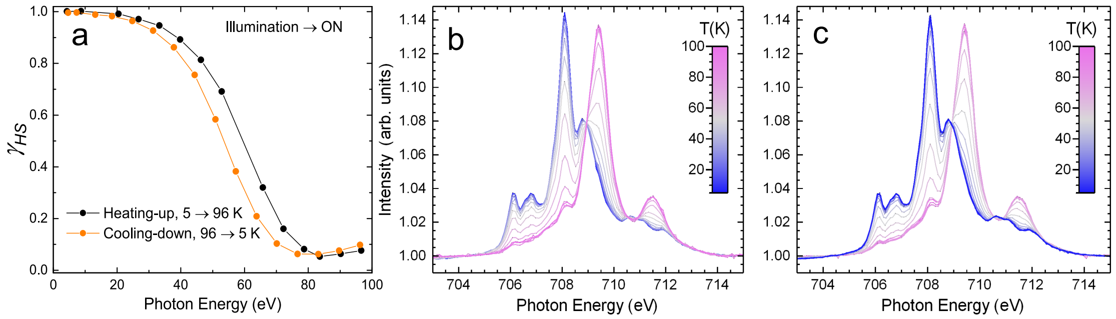

Supplementary Figure 2: Reversible HS⇌LS switching at low temperatures.
(a) Change in γ_{HS} during the heating (black dots) and cooling (orange dots) cycles between 5 and 96 K of 0.69(8) ML of Fe(bpz)-bipy on HOPG (solid lines are a guide to the eyes). The sample has been first heated from 5 to 96 K and then cooled back to 5 K at the rate of $4\,\mathrm{K{\cdot}min^{-1}}$ under continuous illumination by a green LED (light emitting diode, $\lambda = 520\,\mathrm{nm}$) of the same intensity as used for the experiment shown in Figure 3b of the main text. The Fe L_3 spectra recorded during the heating and cooling cycles corresponding to the black and orange dots in (a) are shown in (b) and (c), respectively. Below 40 K, the spin-state is dominated by the light-induced metastable HS state; between 40 and 60 K, there is a rapid interconversion from the HS to the LS state during the heating cycle and from the LS to the HS states during the cooling cycle. For temperatures > 60 K, the LS state becomes dominant due to the thermally activated back-switching from the HS state to the LS ground state. There is a residual HS fraction of about 5% in the thermally-activated regime, as can be directly inferred from the presence of a "bump" at the photon energy of 708.1 eV in (b) and (c) (deep-magenta-coloured spectra). It is due to the soft-x-ray-induced excited spin state trapping (SOXIESST).

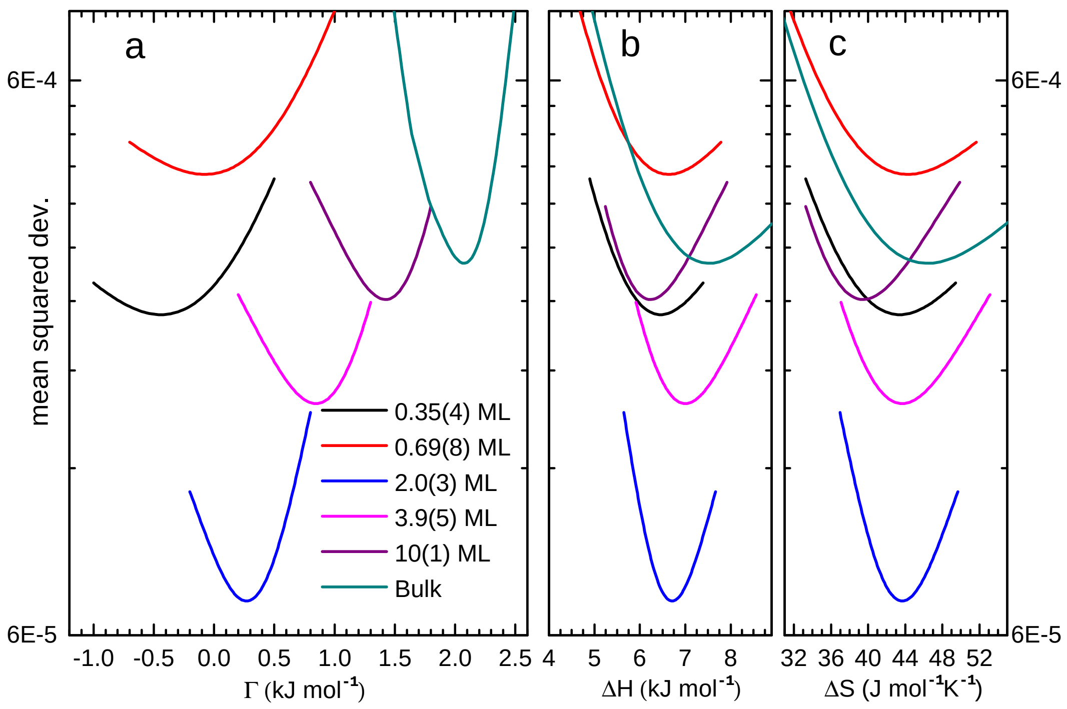

Supplementary Figure 3: Determining the thermodynamic best fit parameters using the Slichter–Drickamer model. Variations in the mean squared deviations (m.s.d.) between the fits of the Slichter–Drickamer model and the experimental data with respect to a variation in one of the parameters — the interaction parameter Γ (a), the enthalpy change ΔH (b), and the entropy change ΔS (c) — while fitting the other two. The minima of m.s.d. of the ultrathin films are similar to that of the bulk sample, or lower in the case of the 2.0(3)- and 3.9(5)-ML samples, indicating the suitability of the model in describing the thermally-induced spin-crossover in ultrathin films. The experimental data for obtaining the bulk fits is taken from Ref. 1.

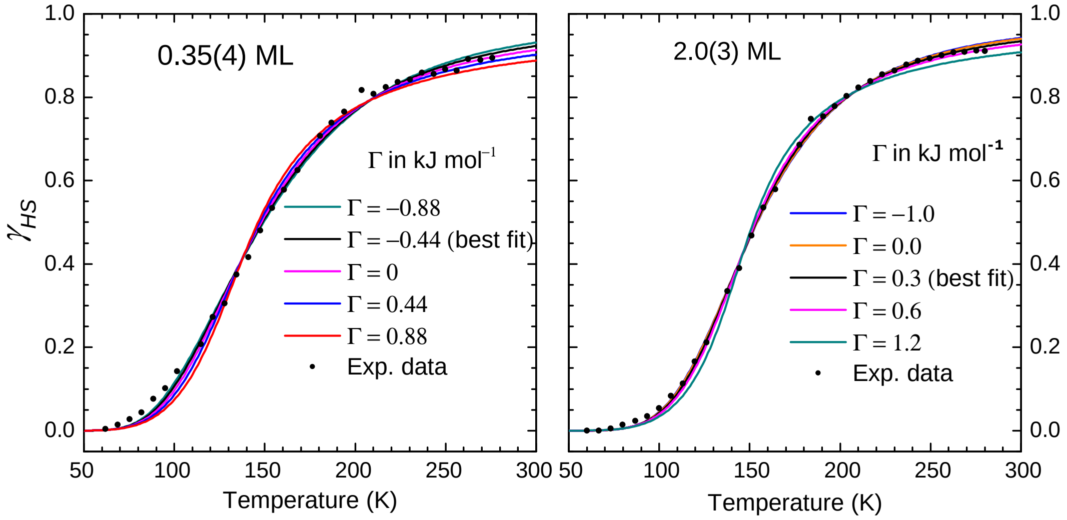

Supplementary Figure 4: Comparative fitting curves against the best fit from the Slichter–Drickamer model. Different fitting curves from the S–D model are plotted by keeping the interaction parameter Γ fixed at certain values — ΔS and ΔH become the variables — in the case of the 0.35(4)-ML sample (left panel) and the 2.0(3)-ML sample (right panel), with the black dots representing the experimental data. The resulting best fit parameters for ΔS and ΔH are listed in Supplementary Table 1. The effect of a higher (lower) value of Γ can be partly compensated by lower (higher) values of ΔH and ΔS. However, the shape of the temperature-dependent spin transition curve does not stay the same as well as the HS content at 300 K. There are clear minima in the mean squared deviation of the fits as a function of Γ, as shown in Supplementary Figure 3.

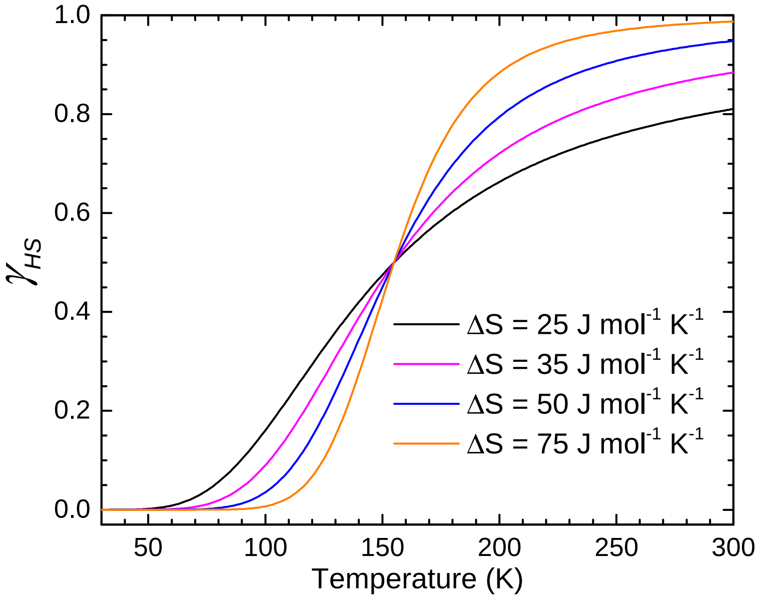

Supplementary Figure 5: Spin-crossover model with no cooperative effects. Calculated curves of the HS fraction as a function of temperature for different values of ΔH and ΔS, keeping the transition temperature $T_{1/2} = \Delta H/\Delta S$ constant at 155 K. The curves have been calculated assuming no cooperativity — $\Gamma = 0$ — which reduces the Slichter-Drickamer model to the van't Hoff model. Different values of ΔH and ΔS lead to different widths of the thermal spin-state transition, but at the same time significantly influence the amount of HS molecules γ_{HS} at room temperature. This is contrary to the experimental result shown in Supplementary Figure 1c, which shows the uniformity in the HS (and LS) compositions across all the samples at RT. Hence, cooperative effects have to be taken into account while describing the thermal-induced spin-crossover phenomenon in the present samples.

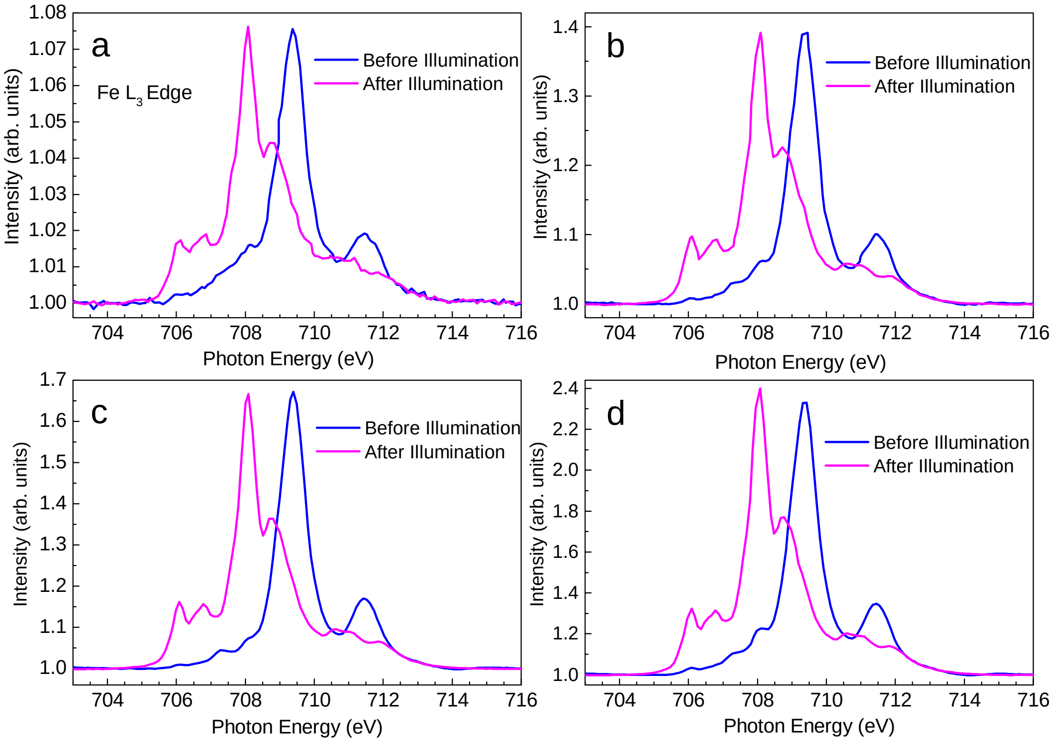

Supplementary Figure 6: Fe L_3 XA spectra before and after illumination. Fe L_3 XA spectra taken before (blue) and after (magenta) illumination for the LIESST experiments at 5 K given in Fig. 3c of the main text; (a) 0.35(4) ML, (b) 2.0(3) ML, (c) 3.9(5) ML, and (d) 10(1) ML. These spectral line shapes are very similar to the ones obtained from multiplet calculations, with the spectra recorded before and after illumination as representing the HS and LS states, respectively [2, 3].

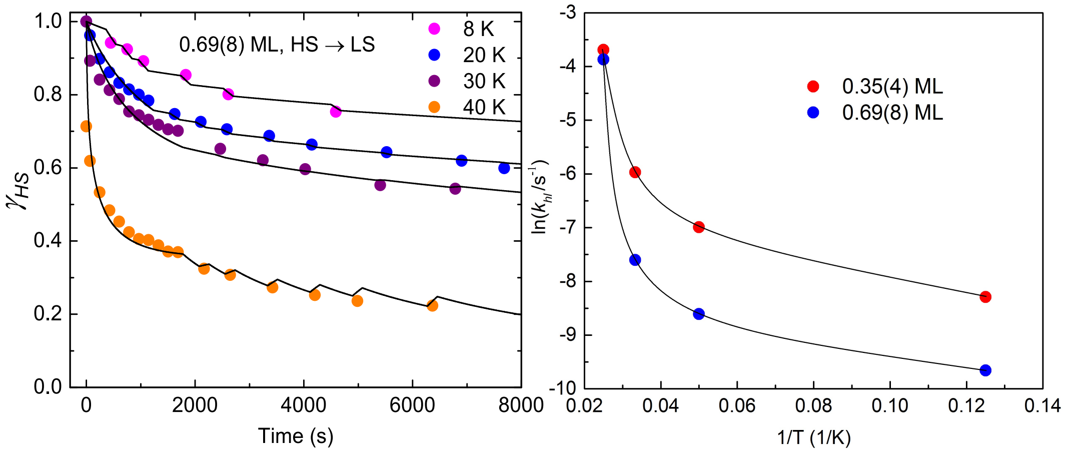

Supplementary Figure 7: HS→LS relaxation and SOXIESST corrections. The left panel shows the HS→LS state relaxation of 0.69(8) ML of Fe(bpz)-bipy on HOPG at low temperatures. The simultaneous fit to Equation (2) of the main manuscript including SOXIESST corrections yields an interaction parameter $\alpha = -6.4(3)$. The step-like features in the fit curves are due to the SOXIESST (LS→HS) and reverse-SOXIESST (HS→LS) contributions in the spin relaxation processes during the recording of the spectra. These contributions were considered in the calculation of the relaxation curves, using values for the SOXIESST (and reverse-SOXIESST) rates from Ref. 4 for a similar sample where the rates were systematically studied at 5 K. It is assumed that the SOXIESST (and reverse-SOXIESST) rates are independent of temperature. The direction of the steps in the left panel and Figure 3c of the main manuscript depends on the amount of the HS or the LS fraction at the instant of measurements: at high HS contents, the x-ray-induced effect is dominated by reverse-SOXIESST resulting in the downward steps, while the reverse is true in the case of high LS contents (SOXIESST domination and upward steps). The height of the steps depends on the "distance" to the SOXIESST saturation spin state of 60% HS [4]. The right panel shows a comparative plot of $\ln(k_{hl}/s^{-1})$ as a function of 1/T (1/K) for the 0.35(4)- and 0.69(8)-ML samples (k_{hl} is the HS→LS relaxation rate constant). Solid lines are guides to the eyes.

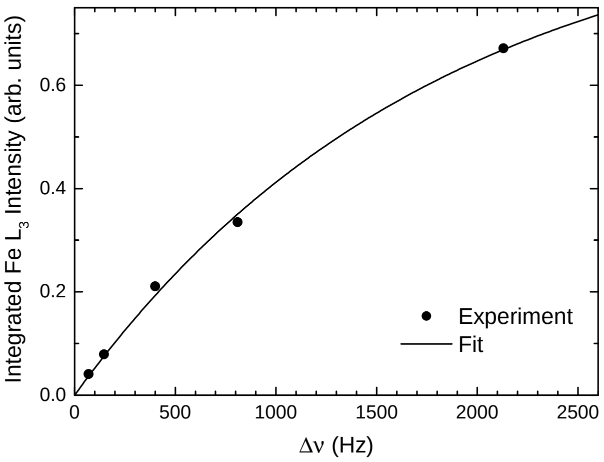

Supplementary Figure 8: Thickness dependence of the absorption signal. The dots represent the integrated Fe L_3 absorption signal as a function of frequency change of the quartz crystal for the samples ranging from 0.35(4) to 10(1) ML. The solid line is the best fit of the data to Equation (3) of Supplementary Notes 1.

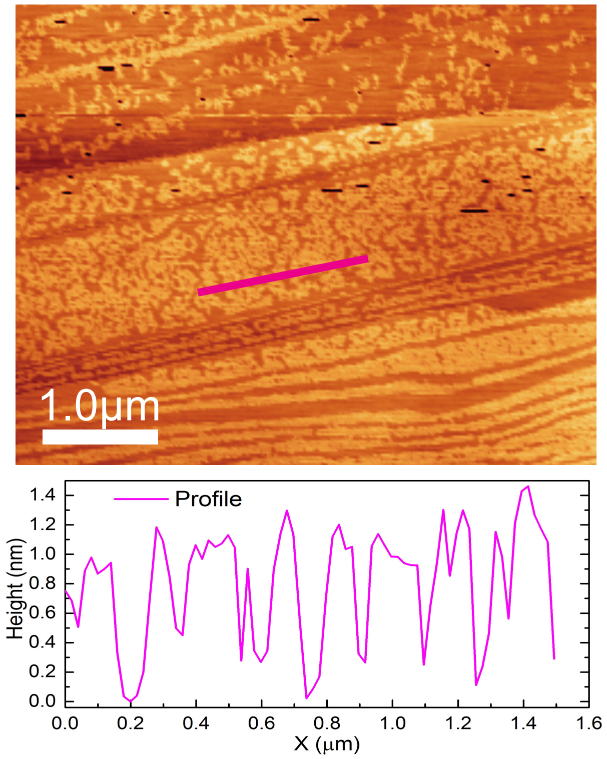

Supplementary Figure 9: AFM image of a submonolayer of Fe(bpz)-bipy on HOPG. The atomic force microscopy image has been recorded under ambient conditions. The bottom panel shows the line profile along the magenta line indicated in the AFM image.

Supplementary Table 1: Values of the fit parameters of Supplementary Figure 4.

Coverage (ML)	Γ^*	ΔH	ΔS
0.35(4)	-0.88	7.2(1)	48.1(8)
	-0.44	6.4(4)	43(3)
	0.0	5.7(1)	38.7(7)
	0.44	5.0(1)	33.9(7)
	0.88	4.3(1)	29.1(9)
2.0(3)	-1.0	9.3(2)	60(1)
	0.0	7.2(1)	47.1(5)
	0.3	6.6(1)	43.4(4)
	0.6	6.0(1)	39.5(4)
	1.2	4.9(1)	32(1)

* Γ and ΔH in kJ mol^{-1}; ΔS in J mol^{-1}K^{-1}

Supplementary Notes 1: Thickness estimation of the molecular layer

The frequency shift of a quartz micro balance integrated into the evaporator was used as a relative measure of the thickness of the molecular layer. The absolute thickness was estimated by comparing the integrated Fe L_3 XAS intensity at low molecular coverage to that of an Fe octaethyl-porphyrin (Cl)/Cu(001) reference sample which had been measured both with STM and XAS [5]. The total electron yield for an adsorbate layer (A) of thickness Δx on a semi-infinite substrate (C) and a gold grid reference (G) is given by [6, 7]:

$$Y_{\text{sample}} \sim \big(1 - \mathrm{e}^{-\frac{\Delta x}{\lambda_e}}\big)\lambda_e \mu_A + \mathrm{e}^{-\frac{\Delta x}{\lambda_e}} \lambda_e \mu_C \ , \quad Y_{\text{grid}} \sim \lambda_e \mu_G \tag{1}$$

assuming that the attenuation length of the secondary electrons λ_e is material-independent and much smaller than that of the x rays ($\lambda_e \ll 1/\mu$). After normalizing to the gold grid, the signal is normalized to the pre-edge absorption coefficient of the adsorbate. Assuming constant absorption of the substrate and grid in the relevant energy range ($\mu_C = \mu_C^{\text{pre}}$, $\mu_G = \mu_G^{\text{pre}}$) yields:

$$Y_{\text{sample/grid}}/Y_{\text{sample/grid}}^{\text{pre}} = \frac{\big(1 - \mathrm{e}^{-\frac{\Delta x}{\lambda_e}}\big)\frac{\mu_A}{\mu_C^{\text{pre}}} + \mathrm{e}^{-\frac{\Delta x}{\lambda_e}}}{\big(1 - \mathrm{e}^{-\frac{\Delta x}{\lambda_e}}\big)\frac{\mu_A^{\text{pre}}}{\mu_C^{\text{pre}}} + \mathrm{e}^{-\frac{\Delta x}{\lambda_e}}} \approx 1 + \frac{\mu_A - \mu_A^{\text{pre}}}{\mu_C^{\text{pre}}}\frac{\Delta x}{\lambda_e} + \dots \tag{2}$$

In the low-coverage regime this signal is proportional to the ratio of the resonance intensity of the adsorbate and the absorption coefficient of the substrate after subtracting unity. To compare the adsorbate signal on different substrates, the ratio of their absorption coefficients must be known. On metals the efficiency of electron extraction from the substrate changes with molecular coverage due to the change in work function. The XA intensity ratio between HOPG and Cu(001) substrates in the pre-edge region of the Fe L_3 edge has been determined by XA measurements under identical conditions with normalization to the gold grid. For clean HOPG and Cu(001) substrates the intensity ratio is 0.84(8) and decreases to 0.65(7) for a coverage of one monolayer. The reference sample has an areal density of 0.14 Fe ions/nm^2. Following Ref. 3 and 8, an areal density of 0.82 Fe ions/nm^2 is assumed for 1 ML. Comparing the integrated Fe L_3 intensity of the two samples with lowest coverage, we obtain a factor of 205(25) Hz/ML that relates the frequency shift to the number of monolayers.
To analyze the thickness dependence of the absorption signal, the pre-edge signal can be subtracted after normalizing to the gold grid. Using the same assumptions as above yields:

$$Y_{\text{sample/grid}} - Y_{\text{sample/grid}}^{\text{pre}} \sim \big(1 - \mathrm{e}^{-\frac{\Delta x}{\lambda_e}}\big)\frac{\mu_A - \mu_A^{\text{pre}}}{\mu_G^{\text{pre}}}. \tag{3}$$

The best fit of this relationship to the experimental data is shown in Supplementary Figure 8. A comparably high value of $\lambda_e = 1780(230)$ Hz $= 8.7(1.6)$ ML for the attenuation length of the electron yield is obtained. This may be attributed to a less dense packing of the atoms in the molecular layer and a lower degree of electronic conjugation compared to metals or graphite.

Supplementary References

[1] Moliner, N. *et al.* Thermal and optical switching of molecular spin states in the [$FeL[H_2B(pz)_2]_2$] spin-crossover system (L = bpy, phen). *J. Phys. Chem. B* **106**, 4276 (2002).

[2] Warner, B. *et al.* Temperature- and light-induced spin crossover observed by x-ray spectroscopy on isolated Fe(II) complexes on gold. *J. Phys. Chem. Lett.* **4**, 1546–1552 (2013).

[3] Bernien, M. *et al.* Highly efficient thermal and light-induced spin-state switching of an Fe(II) complex in direct contact with a solid surface. *ACS Nano* **9**, 8960–8966 (2015).

[4] Kipgen, L. *et al.* Soft-x-ray-induced spin-state switching of an adsorbed Fe(II) spin-crossover complex. *J. Phys.: Condens. Matter* **29**, 394003 (2017).

[5] Herper, H. C. *et al.* Iron Porphyrin Molecules on Cu(001): Influence of Adlayers and Ligands on the Magnetic Properties. *Phys. Rev. B* **87**, 174425 (2013).

[6] Stöhr, J. (ed.) *NEXAFS Spectroscopy*, vol. 25 (Springer-Verlag: Berlin, Heidelberg, 1992).

[7] Nakajima, R., Stöhr, J. & Idzerda, Y. U. Electron-yield saturation effects in L-edge x-ray magnetic circular dichroism spectra of Fe, Co, and Ni. *Phys. Rev. B* **59**, 6421–6429 (1999).

[8] Gopakumar, T. G. *et al.* Spin-crossover complex on Au(111): Structural and electronic differences between mono- and multilayers. *Chem. Eur. J* **19**, 15702–15709 (2013).
