# Supplementary material for: Evolution of cooperativity in the spin transition of an iron(II) complex on a graphite surface
Source: Nat Commun. 2018 Jul 30;9:2984. doi: 10.1038/s41467-018-05399-8 (PMC6065309; doi:10.1038/s41467-018-05399-8)
Supplement: Supplementary file 2 — Peer Review [file 41467_2018_5399_MOESM2_ESM.pdf]

Reviewers' comments:

Reviewer #1 (Remarks to the Author):

The work by Kipgen et al. shows new results on the spin-crossover molecule (SCM) behavior in the limit of submonolayer on a graphite surface up to ultrathin films (8 ML). The main result of the paper is that the spin crossover curve with temperature is thickness dependant, what is interpreted by a change of cooperativity, going from anticooperative effects in the submonolayer range to cooperative (like the bulk compound) for ultrathin films. In general, the work seems reliable, with raw data of very good quality and a thorough analysis, on a topic which is of rather high interest. I think it deserves publication in Nature Communications.

I have nevertheless few comments to improve the quality of the manuscript :

-an important issue of papers dealing with submonolayer SCM is the determination of the coverage. Although an attention is paid on this problem in this paper, I find that it should be explained more clearly. For example, Fig. 1 gives the impression that the calibration is done by comparison with AFM images on the measured sample (what would be the best proof to my opinion). If I understand well the last sentence of the 'Methods' part, it is not the case (I hardly understand why as the AFM measures are done ex situ) and I don't find precise enough 'are prepared in the same manner'. I would therefore remove this AFM image that gives a wrong impression. If one wants to know how the coverage is calibrated, one has to go and read ref 44, that itself refers to its reference 30 (where XAS and STM on a completely different molecule and substrate are shown) ! In ref 30 of 44, I cannot find the definition of a ML (in Fe areal density) so the quantitative comparison is delicate. All in all, I understand that the calibration is done using exclusively the XAS signal (and not a mix or average with a quartz microbalance). If so, I don't understand how the higher thicknesses are determined. Is it assumed that the XAS signal is proportional to the thickness up to 8 ML ? I would have thought that the electron mean free path at this energy is already shorter than that. Be more precise on this part in method and I think that a detailed part in the SI on your calibration procedure can help other teams to compare their results with yours.

-the measurement of Fig. 2a have been done with a ramp of decreasing temperature. Even if it is known the bulk compound shows no hysteretic behaviour, have you measured the increasing temperature ramp to check if this non hysteretic behaviour is also observed for ultrathin samples ? It could also gives you an estimate

of the error bar on your data points. If not, this should be mentioned in the manuscript.

-your interpretation of data of Fig. 2a is a modification of gamma, from negative to positive. Delta H and
Delta S are rather constant. Would it be possible to fit the data with a different value of gamma and different
values of Delta H and Delta S ? It is not obvious for me that those latter should remain unaffected when
the molecules are in contact with a surface. Can you at least discuss the reliability of your fit with three parameters ?

-Fig. 3 c strengthens the presence of anticooperativity. However, to be fully conclusive, you should
present such relaxation curves for samples above the monolayer and show that it is indeed different.

-the steplike behaviour of the fits of Fig. 3c and Fig. S2 is due to SOXIESST effect. I don't understand
steps are higher at higher temperature (even non monotonous with temperature in S2). SOXIESST is generally
far less important at higher temperature. Could you detail this fitting procedure ?

Reviewer #2 (Remarks to the Author):

This manuscript reports thermal and light-induced spin-crossover of a previously reported molecule, evaporated onto a graphite surface. Different degrees of surface coverage are observed, from 0.4 monolayers to 7.5 monolayers, and the variation of the transition cooperativity is traced as the surface coverage increases. This kind of study has been pursued by a number of groups for some years, including these authors, but presents several technical challenges (which are described in the introduction). The innovations that led to the success of this study are the discovery of conditions where the complex consistently retains its integrity on the surface; and, the use of photoelectron spectroscopy to quantitate the spin-crossover transition. This is the clearest and most successful study of this type yet reported.

Although I'm not expert on the technicalities of the surface experiments, the manuscript was easy to read and I found no errors in the interpretation or discussion of the results. I'm happy for this to be published subject to these small comments.

Figure 2 is particularly striking. Since T1/2 and the thermodynamic parameters for the transition show hardly any variation with surface coverage, that implies all the samples should adopt a similar molecular packing. I would add T1/2 as an extra column to Table 1 to emphasize that.

The stretched exponential LIESST relaxation behavior is perfectly reasonable. If nothing else, not all molecules in the films are in the same environment (some are in the bulk and some are in the surface). LIESST relaxation rates depend on the lattice, as well as on individual molecules, so such a dependence is reasonable. It's interesting that the khl values for the 0.8 ML sample in the SI are the same order of magnitude as for the bulk material at the same temperatures (ref. 52). The 0.4 ML sample relaxes more rapidly, which is also to be expected given its larger surface area. I would have made more of that in the discussion.

It's also worth contrasting these results with comparable studies from nanoparticle samples, which show a huge variation in spin-crossover cooperativity and completeness at sub-30 nm particle sizes (reviewed in New J. Chem. 2014, 38, 1834). The difference arises because nanoparticles are always coated with a stabilizer which acts as a rigid matrix, whereas these samples have no such

surface coating and so don’t experience the same matrix effects.

Reviewer #3 (Remarks to the Author):

The authors report experiments in which the spin state of ultrathin SCM films, from submonolayer to several monolayer, is determined using XAS, with the aim of understanding how film dimensionality (sub 2D to 2D to supra 2D) affects cooperativity in driving the spin transition, both thermally and optically. The experiment is well-crafted, and the data analysis/discussion is very insightful. The authors are to be commended for painstakingly attempting to separate the various contributions (cooperativity, temperature, optical/X-ray light) to the actual spin state. Overall, I would recommend publication in Nature Comm. (because this level of insight is deserving) once certain key points are addressed.

Key points
1) Determination of thickness

In the methods section: “The molecular coverage is estimated from the integrated peak intensity of the Fe L3 spectrum. The details of the coverage estimation procedure, making use of the XAS and STM measurements have already been described elsewhere. 44”

The authors make no mention of STM measurements in the present manuscript. Ref. 44 does not appear to have STM measurements either. The present work and Ref. 44 use a XAS absorption edge to determine thickness. This method is only valid, strictly speaking, in the sub-monolayer regime, because the additional ML beyond the first ML will attenuate the signal from the 1st ML, and so on. One could correct with a thickness-dependent attenuation factor, but the authors are not doing so. Please clarify.

Ex-situ AFM data is mentioned on p. 5 and through Fig. 1c. SCO films can be very sensitive to air moisture, which in turn could alter the determination of thickness. Please discuss in the SI the air stability of these films: show the same region scanned in AFM as soon as possible after growth (specify time of exposure to air, humidity conditions etc…), and after 1 hour.

Please improve all figures with error bars for the film thickness. Does this alter the interpretation of results?

2) Claim of complete, reversible transition
The authors mention several times that the HS->LS->HS transition they observe is ‘complete’. This claim isn’t necessary to justify publication, but should be better justified if maintained.

“These spectral line shapes have been established as representative of pure HS and LS states, respectively. 32,44,51”
This referencing isn’t sufficient if the authors wish to pursue this claim. Please compare bulk and ultrathin film spectra in the HS and LS states in a Figure in the SI that is referenced in the main text.

“The HS fraction as a function of illumination time is obtained by normalization of the recorded timescan signal
between 0 (pure LS state) and 1 (pure HS state).“

Then, separately, is the issue of what final state is achieved in LIESST/SOXIESST experiments. What proof is there that the initial and final states of the LIESST/SOXIESST experiment are pure LS and pure HS? This assumption plays a role in the authors’ discussion on separating LIESST and SOXIESST contributions. Same request for a SI Figure.

"The original RT spectrum is fully recovered upon further heating to 300 K, and hence provingthe complete reversibility i n the spin transition."
Same request for a SI Figure: please compare the initial and this spectrum in a SI figure that is referenced in the main text.

3) Claim of tracking cooperativity: fitting procedure
The claim of tracking cooperativity with effective coverage relies on a fitting procedure. To better understand the merits of the least squares fit, including the main manuscript claim of switchover from anticooperative to cooperative behavior:
- Define all variables. Is Eq. 1 in fact Eq. 42 in Ref. 48, such that R is the Boltzmann constant??
- Specify which are dependent/ independent fitting parameters
- Specify the input parameters (eg gammaHS from experimental data?)
- If there are more than two adjustable parameters in the fit (DS DH, DT, Gamma) , please justify the correctness of the fit. One approach is to show that obtaining positive Gamma for sub-monolayer coverage would be a wrong fit statistically.
- In Table I, specify what the numbers in parentheses in the Gamma column are.

4) Claim of use toward designing SCM-based devices
The manuscript mentions several times that these results will 'serve as useful yardsticks in designing SCM-based devices." If the device in mind contains metallic electrodes, then much of the observations reported here (swichover from anticooperative to cooperative behavior) would be overshadowed by charge transfer. It would be useful to help the reader here by explaining what one could expect to happen when replacing HOPG with a noble or transition metal used within a device . In this context, a good reference of SCM properties when deposited onto noble and transition metal surfaces could be 10.1063/1.4973511 . Or perhaps the authors are referring to non-metallic contacts (e.g. graphene?)

- Misc
"Regardless of the mechanism, it should be mentioned that this is the first report on the HS!LS relaxation of SCMs on a surface exhibiting a stretched exponential decay"
What about the data of Fig. 3i and 4g from Ref. 42 (Bairagi Nat Comm 2016)?

- Mechanics
- Fig 1e: unless one can anticipate the results, the panel's many overlapping data make it hard to discern. Colors in Fig. 1e are hard to follow (red vs. orange). Overlap of data makes it hard to follow: markers to point to data?
- Fig 1 e and text: please do not presuppose that the LT + light spectra are HS spectra as denoted: label them as LT data with light on, and argue in text that the spectrum is that of the HS state.
- "Since all the curves can be fitted by a single exponential," : The sentence is misleading: a function of the form 'single exponential' was used to fit all curves each with a different rate constant. One could read that a single function, i.e. an exponential with the same rate constant, was used. Please correct.

"It yields a gradual evolution in cooperative spin transition in going from submono-
149 layers to multilayers: negative interaction parameters at 0.4(1) and 0.8(1) ML, positive in
150 2.3(2) ML, and further increasing with increasing coverage."
Please refer to Gamma here.

- Minor English:
"accompanied with the generation" ->"accompanied by the generation"
"shows such an AFM" ->shows such an AFM "shows one such AFM"

Authors' response.

We are glad to learn that all three Reviewers find our manuscript of sufficient interest for publication in Nature Communications. We thank the Reviewers for the careful reading and for drawing our attention to certain points that were not clear enough in the previous version of the manuscript. We have addressed all those points, as outlined in the following, which helped to improve the manuscript. We hope that it is now ready for publication in Nature Communications.

Sincerely,

Lalminthang Kipgen (on behalf of all authors)

Reviewer #1:

The work by Kipgen et al. shows new results on the spin-crossover molecule (SCM) behavior in the limit of submonolayer on a graphite surface up to ultrathin films (8 ML). The main result of the paper is that the spin crossover curve with temperature is thickness dependent, what is interpreted by a change of cooperativity, going from anticooperative effects in the submonolayer range to cooperative (like the bulk compound) for ultrathin films. In general, the work seems reliable, with raw data of very good quality and a thorough analysis, on a topic which is of rather high interest. I think it deserves publication in Nature Communications.

I have nevertheless few comments to improve the quality of the manuscript:

-an important issue of papers dealing with submonolayer SCM is the determination of the coverage. Although an attention is paid on this problem in this paper, I find that it should be explained more clearly. For example, Fig. 1 gives the impression that the calibration is done by comparison with AFM images on the measured sample (what would be the best proof to my opinion). If I understand well the last sentence of the 'Methods' part, it is not the case (I hardly understand why as the AFM measures are done ex situ) and I don't find precise enough 'are prepared in the same manner'. I would therefore remove this AFM image that gives a wrong impression. If one wants to know how the coverage is calibrated, one has to go and read ref 44, that itself refers to its reference 30 (where XAS and STM on a completely different molecule and substrate are shown)! In ref 30 of 44, I cannot find the definition of a ML (in Fe areal density) so the quantitative comparison is delicate. All in all, I understand that the calibration is done using exclusively the XAS signal (and not a mix or average with a quartz microbalance). If so, I don't understand how the higher thicknesses are determined. Is it assumed that the XAS signal is proportional to the thickness up to 8 ML? I would have thought that

the electron mean free path at this energy is already shorter than that. Be more precise on this part in method and I think that a detailed part in the SI on your calibration procedure can help other teams to compare their results with yours.

We thank the Reviewer for these valuable suggestions; accordingly, the AFM image is now moved to the supplementary information (SI). We felt the need to include the ex-situ AFM image mainly to address the question of crystallite formation (along similar lines already explicitly stated in the previous version of the manuscript). It has nothing to do with the thickness estimation. The thickness estimation was done exclusively based on the XAS signal using reference values from our previously published works. We agreed with and took on-board the Reviewer's suggestion to not ignore the attenuations of the total electron yield signal at higher thicknesses. Now, the thicknesses are estimated based on the XAS signal at submonolayer coverages, comparing to simultaneous STM–XAS calibration measurements of a different Fe-containing molecule, taking into account the different areal density of molecules and the different background contribution of the substrate. Information on the total-electron-yield attenuation in the present system and the thickness at multilayer coverages comes from the frequency change of the quartz crystal attached to the evaporator in conjunction with the XAS signal and an equation describing the attenuation of electrons. These are now described in detail in the SI. For up to about 4 ML, the previous and the revised estimates deviate only slightly, while for the thickest film the attenuation of the total electron yield cannot be neglected and leads to a thickness of 10 ML instead of 7.5 ML as in the previous estimate.

-the measurement of Fig. 2a have been done with a ramp of decreasing temperature. Even if it is known the bulk compound shows no hysteretic behaviour, have you measured the increasing temperature ramp to check if this non hysteretic behaviour is also observed for ultrathin samples? It could also gives you an estimate of the error bar on your data points. If not, this should be mentioned in the manuscript.

No hysteresis measurements are done in these samples, partly due to the limited time allotted to us for these measurements, and partly due to the absence of hysteresis even in the case of the bulk. However, it is worth pointing out that vacuum-deposited thin films of Fe(bpz)-bipy have been reported to exhibit a small hysteresis (about 4 K).

Text added to the manuscript:

(Line # 292—298) "The existence of hysteretic behavior in these ultrathin films of Fe(bpz)-bipy is not known, as the spin-state change in the opposite direction, *i.e.*, from low- to high-temperature ramp-up, has not been measured. A small hysteresis of about 4 K has been reported in a relatively thick vacuum-deposited film (355 nm) of Fe(bpz)-bipy [31], despite the absence of such a behaviour in the bulk [54,66]. To the best of our knowledge, for vacuum-deposited films with thickness in the range of

our samples – maximum thickness of about 12 nm – the presence of hysteresis has never been reported."

-your interpretation of data of Fig. 2a is a modification of gamma, from negative to positive. Delta H and Delta S are rather constant. Would it be possible to fit the data with a different value of gamma and different values of Delta H and Delta S? It is not obvious for me that those latter should remain unaffected when the molecules are in contact with a surface. Can you at least discuss the reliability of your fit with three parameters?

The Reviewer is right that the values of ΔH and ΔS do not necessarily need to be the same in contact with a surface. This is indeed what comes out of the fits. In a new Fig. S3 in the SI we plot the mean squared deviation between fit and experiment as a function of one of the parameters while fitting the others, including that of the bulk molecule. Although there exists a certain correlation between parameters in the S–D model, there is a clear minimum in the mean squared deviation for each of the parameters when fitting the others.

While a variation of ΔH and ΔS also leads to a change in the thermal transition width of the spin transition, the shape of the curves is distinctly different from that including cooperativity. In Supplementary Fig. S4 we show calculated transition curves with the interaction parameter $\Gamma = 0$ for different values of ΔH and ΔS. As can be seen, a change in the width of the curves is accompanied by a different amount of HS and LS at RT, which is not observed in the experiment. In Supplementary Fig. S1c we compare the Fe L_3 XA spectra of all samples at RT. Clearly, no variation of the HS–LS composition is observed and a fit to the experimental data as shown in Fig. 2a is thus only possible by including cooperativity. As suggested by the Reviewer, in Supplementary Fig. S5 we show the best fits with different fixed values of Γ, ΔH, and ΔS in the case of the 0.35(4) and 2.0(3)-ML samples.

-Fig. 3 c strengthens the presence of anticooperativity. However, to be fully conclusive, you should present such relaxation curves for samples above the monolayer and show that it is indeed different.

Unfortunately, we don't have such measurements at higher coverages – which are rather time-consuming. However, a distribution in the energy barrier between the HS and LS states – which may arise from disorder or conformational flexibility in the ligands and could be more pronounced at higher coverages, where the films are expected to be more amorphous – will also contribute towards a stretched-exponential behaviour of the relaxation curve. This is reported to be the case in amorphous SCMs that exhibit both strong cooperative temperature-induced spin transition and a stretched-exponential spin relaxation [64, 65].

So, the observed stretched exponential curves are not unequivocally proving anticooperative spin relaxation. (Already stated in the original MS.)

To further emphasize the complexities involved in the HS→LS relaxation, we have expanded the corresponding discussion in line # 239—249 and # 264—269; also, please refer to Reviewer #2 – second comment.

(Line # 239—249) "In comparison to the 0.69(8)-ML sample, the metastable HS state of the 0.35(4)-ML sample decays much more rapidly to the ground state (LS state) in the temperature range between 10 and 30 K. However, at 40 K, the relaxation rates become similar: 0.025(5) s^{-1} and 0.021(7) s^{-1} for the 0.35(4) and 0.69(8)-ML sample, respectively. In the bulk relaxation data provided in Ref. 54, the relaxation rate at 42 K is about three orders of magnitude lower than that of the submonolayer samples at 40 K. The differences in the decay rates in the submonolayer samples as compared to the bulk might arise from an enhanced tunneling rate, a reduction in the energy barriers between the HS and LS wells, or from a combination of both. (The quantum tunneling rate is related to the vibrational frequency of the Fe[N6] core [62].)"

(line # 264—269) "Alternatively, a distribution in the energy barriers between HS and LS states—arising from disorder or conformational flexibility of the ligand—can also result in a stretched exponential HS→LS relaxation, as reported in the case of some bulk SCMs exhibiting strong cooperativity in the temperature-induced spin transition, but nevertheless displaying a stretched-exponential spin relaxation due to such factors [64,65]. It is therefore not clear whether the stretched exponential decay results exclusively from an antagonistic (or anticooperative) spin transition."

-the steplike behaviour of the fits of Fig. 3c and Fig. S2 is due to SOXIESST effect. I don't understand steps are higher at higher temperature (even non monotonous with temperature in S2). SOXIESST is generally far less important at higher temperature. Could you detail this fitting procedure?

(Fig. S2 is now Fig. S7 in the revised SI.)

In our fitting procedure we assume that the SOXIESST production rate during the XA measurements is independent from temperature, and that it is only the enhanced thermal relaxation of the HS→LS state that makes SOXIESST appear less important at higher temperatures. The x-ray-induced effects are a competition between two processes: LS→HS conversions (SOXIESST) which are dominant at higher LS contents, resulting in "upward steps" and HS→LS conversions (reverse-SOXIESST), which are dominant at higher HS contents, resulting in "downward steps" in Fig3c and Fig. S7. The height of the steps depends on the "distance" to the SOXIESST saturation spin state of 60% HS [53]. We have now added a section in the Supplementary Information explaining the fit in more detail.

Reviewer #2:

This manuscript reports thermal and light-induced spin-crossover of a previously reported molecule, evaporated onto a graphite surface. Different degrees of surface coverage are observed, from 0.4 monolayers to 7.5 monolayers, and the variation of the transition cooperativity is traced as the

surface coverage increases. This kind of study has been pursued by a number of groups for some years, including these authors, but presents several technical challenges (which are described in the introduction). The innovations that led to the success of this study are the discovery of conditions where the complex consistently retains its integrity on the surface; and, the use of photoelectron spectroscopy to quantitate the spin-crossover transition. This is the clearest and most successful study of this type yet reported.

Although I'm not expert on the technicalities of the surface experiments, the manuscript was easy to read and I found no errors in the interpretation or discussion of the results. I'm happy for this to be published subject to these small comments.

Figure 2 is particularly striking. Since $T_{1/2}$ *and the thermodynamic parameters for the transition show hardly any variation with surface coverage, which implies all the samples should adopt a similar molecular packing. I would add* $T_{1/2}$ *as an extra column to Table 1 to emphasize that.*

The $T_{1/2}$ values for all the coverages are added to Table 1.

The stretched exponential LIESST relaxation behavior is perfectly reasonable. If nothing else, not all molecules in the films are in the same environment (some are in the bulk and some are in the surface). LIESST relaxation rates depend on the lattice, as well as on individual molecules, so such a dependence is reasonable. It's interesting that the khl values for the 0.8 ML sample in the SI are the same order of magnitude as for the bulk material at the same temperatures (ref. 52). The 0.4 ML sample relaxes more rapidly, which is also to be expected given its larger surface area. I would have made more of that in the discussion.

We thank the Reviewer for the suggestion. In the case of the 0.4-ML sample (now 0.35(4) ML in the revised thickness estimate), the relaxation from the metastable HS state to the ground state (LS state) in the temperature range of 8–30 K is much more rapid than that of the 0.8-ML sample (now 0.69(8) ML), as the Reviewer has correctly pointed out. At 40 K, the relaxation rates become almost the same for both samples (0.025(5) and 0.021(7) s^{-1} for the 0.35(4) and 0.69(8)-ML sample, respectively). However, at the comparable temperatures of 35 and 42 K (if these are the temperatures the Reviewer is referring to), the relaxation rates of the bulk are about three orders of magnitude lower than that of the submonolayers at 40 K [54]. We thus disagree with the Reviewer that the decay rates of the submonolayers and the bulk are of similar order of magnitude in the region of 40 K. Having stated that, we would like to add to the manuscript some discussion pointing out the difference in the relaxation rates between the submonolayers and the bulk in the 40-K temperature range and attributing it to the possible difference in the tunneling rates and the energy barriers of the HS and LS wells between the two. We agree with the Reviewer that the stretched-exponential decay could very

well arise from a distribution in activation energies and conformational flexibility of the ligands (as already mentioned in the original manuscript).

The following text has been added to the manuscript to further emphasize the complexities involved in the relaxation process (also please refer to Reviewer #1 – 3rd comment):

(Line # 239—249) "In comparison to the 0.69(8)-ML sample, the metastable HS state of the 0.35(4)-ML sample decays much more rapidly to the ground state (LS state) in the temperature range between 10 and 30 K. However, at 40 K, the relaxation rates become similar: 0.025(5) s^{-1} and 0.021(7) s^{-1} for the 0.35(4) and 0.69(8)-ML sample, respectively. In the bulk relaxation data provided in Ref. 54, the relaxation rate at 42 K is about three orders of magnitude lower than that of the submonolayer samples at 40 K. The differences in the decay rates in the submonolayer samples as compared to the bulk might also arise from an enhanced tunneling rate, a reduction in the energy barriers between the HS and LS wells, or from a combination of both (the quantum tunneling rate is related to the vibrational frequency of the Fe[N6] core [62])."

It's also worth contrasting these results with comparable studies from nanoparticle samples, which show a huge variation in spin-crossover cooperativity and completeness at sub-30 nm particle sizes (reviewed in New J. Chem. 2014, 38, 1834). The difference arises because nanoparticles are always coated with a stabilizer which acts as a rigid matrix, whereas these samples have no such surface coating and so don't experience the same matrix effects.

We thank the Reviewer for pointing this out. The following text has been added to the manuscript based on this suggestion.

(Line # 284—298) "It is interesting to compare the results presented here with spin-crossover nanoparticles, although the direct external environments of SCMs on the surface and that of nanoparticles are different in that the nanoparticles are always coated with a stabilizer which acts as a rigid matrix. Nevertheless, spin-crossover nanoparticles are also found to exhibit a gradual spin-transition curve like the one reported here in the case of ultrathin films, with the transition temperature being proportional to the particles' size. However, at particle sizes smaller than 10 nm, hysteretic behaviour (memory effects) appears – which has been attributed to an increase in the lattice stiffness leading to greater cooperative effects [65]. The existence of hysteretic behavior in these ultrathin films of Fe(bpz)-bipy is not known, as the spin-state change in the opposite direction, i.e., from low- to high-temperature ramp-up, has not been measured. A small hysteresis of about 4 K has been reported in a relatively thick vacuum-deposited film (355 nm) of Fe(bpz)-bipy [31], despite the absence of such a behaviour in the bulk [54,66]. To the best of our knowledge, for vacuum-deposited films with thickness in the range of our samples – maximum thickness of about 12 nm – the presence of hysteresis has never been reported."

Reviewer #3:

The authors report experiments in which the spin state of ultrathin SCM films, from submonolayer to several monolayer, is determined using XAS, with the aim of understanding how film dimensionality (sub 2D to 2D to supra 2D) affects cooperativity in driving the spin transition, both thermally and optically. The experiment is well-crafted, and the data analysis/discussion is very insightful. The authors are to be commended for painstakingly attempting to separate the various contributions (cooperativity, temperature, optical/X-ray light) to the actual spin state. Overall, I would recommend publication in Nature Comm. (because this level of insight is deserving) once certain key points are addressed.

1) Determination of thickness

In the methods section: "The molecular coverage is estimated from the integrated peak intensity of the Fe L_3 spectrum. The details of the coverage estimation procedure, making use of the XAS and STM measurements have already been described elsewhere [44]."

The authors make no mention of STM measurements in the present manuscript. Ref. 44 does not appear to have STM measurements either. The present work and Ref. 44 use a XAS absorption edge to determine thickness. This method is only valid, strictly speaking, in the sub-monolayer regime, because the additional ML beyond the first ML will attenuate the signal from the 1st ML, and so on. One could correct with a thickness-dependent attenuation factor, but the authors are not doing so. Please clarify.

We thank the Reviewer for raising this issue; in fact, it has been raised by Reviewer #1, too. Considering the attenuation of the total electron yield signal results in a correction to the film thickness at higher coverages. We provide the details of the thickness estimation procedure in the Supplementary Information in section S7. (Also, please refer to our response to Reviewer #1 – 1st comment.)

Ex-situ AFM data is mentioned on p. 5 and through Fig. 1c. SCO films can be very sensitive to air moisture, which in turn could alter the determination of thickness. Please discuss in the SI the air stability of these films: show the same region scanned in AFM as soon as possible after growth (specify time of exposure to air, humidity conditions etc…), and after 1 hour.

The thicknesses weren't estimated from the AFM image (if the Reviewer was under this impression). The reference thickness for the XAS was taken from our previously published works. The idea of presenting the AFM (as already stated in our response to Reviewer #1 – 1st comment) had more to do with addressing crystallite formation than anything else. The AFM image is now moved to the Supplementary Information, as suggested by Reviewer #1.

Editorial Note: The AFM image has since been moved to the main manuscript.

Please improve all figures with error bars for the film thickness. Does this alter the interpretation of results?

The determination of the film thicknesses has been refined and is now presented in detail in the Supplementary Information. We now indicate error bars for the film thickness in all figures. The previous and the revised thickness estimates remained almost the same for up to about 4 ML. Our interpretation of results still remains the same.

2) Claim of complete, reversible transition

The authors mention several times that the HS->LS->HS transition they observe is 'complete'. This claim isn't necessary to justify publication, but should be better justified if maintained.
"These spectral line shapes have been established as representative of pure HS and LS states, respectively. 32, 44, 51"
This referencing isn't sufficient if the authors wish to pursue this claim. Please compare bulk and ultrathin film spectra in the HS and LS states in a Figure in the SI that is referenced in the main text.

Following the request of the referee, we have now included the RT and LT (78 K) Fe L_3 XA spectra of the bulk and its comparison with that of the 10(1)-ML sample in the Supplementary Fig. S1 (middle panel).

Text added to the MS:

(Line # 120—124) "The bulk Fe L_3 spectra recorded at RT and LT also yielded correspondingly similar line shapes to the ones described above. (*cf.* Supplementary Fig. S1). While SOXIESST is saturated at about 60% HS [53], the final states in LIESST are assumed to be 100% HS following Ref. 45, where the spectral profiles can be reproduced by multiplet calculations."

(Line # 141—144) "….this number is also reproduced by using the LT spectrum after LIESST (100% HS) as the reference HS state. The presence of a certain quantity of LS molecules at RT in thin films of Fe(bpz)-bipy—similar to the bulk—has also been reported elsewhere [31]."

"The HS fraction as a function of illumination time is obtained by normalization of the recorded timescan signal between 0 (pure LS state) and 1 (pure HS state)."

Then, separately, is the issue of what final state is achieved in LIESST/SOXIESST experiments. What proof is there that the initial and final states of the LIESST/SOXIESST experiment are pure LS and pure HS? This assumption plays a role in the authors' discussion on separating LIESST and SOXIESST contributions. Same request for a SI Figure.

We have dealt with the x-ray induced effects in Ref. 53. While SOXIESST is saturated at about 60% HS, the final states in LIESST are assumed to be 100% HS following Ref. 45, where the spectral profiles can be reproduced by multiplet calculations. The spectra for all the LIESST measurements presented in Fig. 3b are now been provided as Supplementary Fig. S6.

"The original RT spectrum is fully recovered upon further heating to 300 K, and hence proving the complete reversibility in the spin transition."

Same request for a SI Figure: please compare the initial and this spectrum in a SI figure that is referenced in the main text.

On the question of reversible spin switching, a systematic record of the spin transition has been carried out between 5 and 96 K under continuous illumination, for the 0.69(8) ML sample as proof of concept (not included in the previous version of the manuscript). However – despite wrongfully implied by the original text regarding reversibility of the spin-state transition (line # 112—114, reproduced by the Reviewer above) – no such systematic measurements have been carried out for the low- to room-temperature ramp-up due to the limited time allocated for the measurements. We apologize for the error. In the revised manuscript, the issue of the reversibility in the spin transition is addressed through the combined light and temperature effect in the 5 – 96 K range by the following text, along with Fig. S2 of the Supplementary Information:

(Line # 127—132) "To test the reversibility in the spin switching during the cooling and heating cycles, systematic measurements to track the change in Fe L_3 spectra (and hence the change in spin state) are carried out between 5 and 96 K under continuous illumination, for the 0.69(8)-ML sample as proof of concept. This is shown in Supplementary Figure S2: the sample exhibits a near-complete reversible spin transition from HS to LS state during temperature ramp-up and LS to HS state during ramp-down in the aforementioned temperature range."

3) Claim of tracking cooperativity: fitting procedure

The claim of tracking cooperativity with effective coverage relies on a fitting procedure. To better understand the merits of the least squares fit, including the main manuscript claim of switchover from anticooperative to cooperative behavior:

- Define all variables. Is Eq. 1 in fact Eq. 42 in Ref. 48, such that R is the Boltzmann constant??

- Specify which are dependent/ independent fitting parameters

- Specify the input parameters (eg γ_{HS} from experimental data?)

- If there are more than two adjustable parameters in the fit (ΔS, ΔH, ΔT, Γ), please justify the correctness of the fit. One approach is to show that obtaining positive Gamma for sub-monolayer coverage would be a wrong fit statistically.

Please see also our response to Reviewer #1 – 3rd comment.

On the question of the reliability of the fits, we took the Reviewer's suggestion on-board and plotted the transition curves of 0.35(4) and 2.0(3) ML for various values of γ_{HS} around the best fits (*cf.* Supplementary Fig. S5). In addition, the variations in the mean squared deviations with the fitting parameters for all the samples (including the bulk) are given in Supplementary Fig. S3. We now define all the variables in Eq. 1 and specify that Γ, ΔH, and ΔS are the three fitting parameters. ΔT (transition width) is not a fit parameter. The values of ΔT given in Table 1 are determined from the experimental data and calculated from the best fit of the model, respectively. These are now indicated in the footnotes to the table.

Text added to the manuscript.

(Line # 168—173) "R is the universal gas constant. The input or the experimental data in equation (1) is the HS fraction γ_{HS}. Of the three fitting parameters, namely, Γ, ΔH and ΔS; ΔH and ΔS are related by the transition temperature $T_{1/2}$ (defined as the temperature where the population of HS and LS species are equal), as ΔH = ΔS* $T_{1/2}$. If Γ = 0, then equation (1) reduces to the van't Hoff's model (system of non-interacting molecules)."

- In Table I, specify what the numbers in parentheses in the Gamma column are.

The asymmetric errors in Table 1 of the previous version were an attempt to account for the asymmetry in the goodness of fit. Instead, we now give the standard errors for the three fitting Γ, ΔH, and ΔS as obtained from the fit of the non-linear model.

(Footnote Table 1) "The uncertainties of Γ, ΔH, and ΔS given in parentheses are the standard errors as obtained from the fit of the non-linear model. The uncertainties of ΔT and $T_{1/2}$ are estimated from the scattering of the data points."

4) Claim of use toward designing SCM-based devices

The manuscript mentions several times that these results will 'serve as useful yardsticks in designing SCM-based devices." If the device in mind contains metallic electrodes, then much of the observations reported here (switchover from anticooperative to cooperative behavior) would be overshadowed by charge transfer. It would be useful to help the reader here by explaining what one could expect to happen when replacing HOPG with a noble or transition metal used within a device. In this context, a good reference of SCM properties when deposited onto noble and transition metal surfaces could be 10.1063/1.4973511. Or perhaps the authors are referring to non-metallic contacts (e.g. graphene?)

We were indeed intending to refer to materials like graphene or 2D dichalcogenides.

Text added to the manuscript:

(Line # 78—83) "Future devices may likely involve integrating multi-functional molecules like SCMs with 2D materials exhibiting novel properties. In this regard, graphene is among the material of choice

due to its robust optical, electrical and mechanical properties [49]. Understanding the behaviour of SCMs on a highly-oriented pyrolytic graphite surface (HOPG) will be an ideal platform towards integrating it with graphene.″

- Misc

"Regardless of the mechanism, it should be mentioned that this is the first report on the HS→LS relaxation of SCMs on a surface exhibiting a stretched exponential decay"

What about the data of Fig. 3i and 4g from Ref. 42 (Bairagi Nat Comm 2016)?

We are also aware that the decay curves in that paper indeed look like stretched exponentials, but there is a certain ambiguity to it as the authors made no mention/discussion about it being a stretched exponential or of being composed of more than one exponential function.

- Mechanics

- Fig 1e: unless one can anticipate the results, the panel's many overlapping data make it hard to discern. Colors in Fig. 1e are hard to follow (red vs. orange). Overlap of data makes it hard to follow: markers to point to data?

We thank the Reviewer for pointing out these inconveniences; the RT and LT spectra are now plotted in separate panels and the colourings changed for better clarity.

- Fig 1 e and text: please do not presuppose that the LT + light spectra are HS spectra as denoted: label them as LT data with light on, and argue in text that the spectrum is that of the HS state.

These have been changed accordingly.

- "Since all the curves can be fitted by a single exponential," The sentence is misleading: a function of the form 'single exponential' was used to fit all curves each with a different rate constant. One could read that a single function, i.e. an exponential with the same rate constant, was used. Please correct.

It has now been changed along similar lines as suggested by the Reviewer, as Line #211) "…each of the curves can be fitted by a mono-exponential function albeit with different rate constants,…."

"It yields a gradual evolution in cooperative spin transition in going from submono-layers to multilayers: negative interaction parameters at 0.4(1) and 0.8(1) ML, positive in 2.3(2) ML, and further increasing with increasing coverage."

Please refer to Gamma here.

This suggestion has been incorporated, as (Line #176—177) "…negative interaction parameters at 0.35(4) (Γ = -0.44(0.23) kJ mol^{-1}) and 0.69(8) ML (Γ= -0.1(2) kJ mol^{-1}), positive in 2.0(3) ML (Γ = 0.3(1) kJ mol^{-1}), ……"

- Minor English:

"accompanied with the generation" -> *"accompanied by the generation"*

"shows such an AFM" -> *shows such an AFM "shows one such AFM"*

These have been changed as suggested.

REVIEWERS' COMMENTS:

Reviewer #1 (Remarks to the Author):

The authors have taken a great care to answer thoroughly to my questions and comments. I find this work very clean and of high interest for the community working on functional molecular devices. I therefore recommend its publication.

Reviewer #3 (Remarks to the Author):

The authors have answered all questions satisfactorily. The paper is now ready for publications.
